# Methods and Systems of Resistance Training: A Narrative Review Based on Contemporary Recommendations

**DOI:** 10.3390/sports14080348

**Published:** 2026-08-11

**Authors:** Manoel J. Rios, Francisco A. Ferreira, Dale W. Chapman, Ricardo J. Fernandes, Victor Machado Reis

**Affiliations:** 1 Piaget Research Center for Ecological Human Development, Higher School of Sport and Education, Jean Piaget Polytechnic Institute of the North, 4405-678 Vila Nova de Gaia, Portugal; 2 Centre of Research, Education, Innovation and Intervention in Sport and Porto Biomechanics Laboratory, Faculty of Sport, University of Porto, 4200-450 Porto, Portugal; up202100039@edu.fade.up.pt (F.A.F.); ricfer@fade.up.pt (R.J.F.); 3 Curtin School of Allied Health, Curtin University, Perth 6102, Australia; dale.chapman@curtin.edu.au; 4 Department of Sport Sciences, Exercise and Health, University of Trás-os-Montes e Alto Douro, 5001-801 Vila Real, Portugal; victormachadoreis@gmail.com; 5 Research Center in Sports Sciences, Health Sciences and Human Development, 5001-801 Vila Real, Portugal

**Keywords:** resistance training, hypertrophy, strength, training methods, training volume, exercise prescription, neuromuscular adaptations

## Abstract

The purpose of this narrative review was to provide a conceptual and applied framework that distinguishes resistance training methods, strategies, and systems and clarifies how these constructs organize established training variables within contemporary recommendations for exercise prescription. The historical, conceptual, and theoretical synthesis was not restricted by publication date and included seminal studies and previous evidence syntheses. In addition, a focused descriptive mapping of primary experimental studies published between January 2021 and April 2026 was conducted using PubMed, Scopus, and Web of Science. This mapping comprised 40 studies assessing acute responses or chronic adaptations to methods including drop sets, rest–pause, pyramidal loading, eccentric training, blood flow restriction, cluster sets, and velocity-based training. Several studies reported no statistically significant between-method differences in selected strength or hypertrophy outcomes; however, their generally small samples and the absence of equivalence or non-inferiority analyses preclude conclusions of true equivalence. Individual studies also reported context-specific differences in session efficiency, fatigue distribution, movement velocity, regional hypertrophy, and contraction-specific adaptations. Given the heterogeneity of the mapped studies and the absence of formal risk-of-bias or certainty-of-evidence assessments, these observations should be interpreted as descriptive and exploratory rather than as evidence of general comparative superiority. Taken together with the broader historical and synthesized literature, the findings suggest that resistance training adaptations are primarily governed by foundational prescription variables, including load, volume, frequency, effort, density, and exercise selection. The principal contribution of this review is therefore to clarify how methods, strategies, and systems can be selected and organized according to specific goals, individual characteristics, and practical constraints.

## 1. Introduction

Resistance training is widely recognized as one of the most effective interventions for improving skeletal muscle function, promoting increases in strength, hypertrophy, power, and overall physical performance [1,2,3]. These adaptations are mediated by complex physiological mechanisms, including enhanced motor unit recruitment, increased muscle protein synthesis, and structural remodeling of muscle tissue [4]. At a fundamental level, these processes are governed by the manipulation of key training variables, particularly load (intensity [5,6]), volume, frequency, density, and exercise selection. The interaction among these variables determines the magnitude and specificity of the stimulus imposed on skeletal muscle [7,8], ultimately shaping the adaptive response.

In applied practice, these variables are rarely manipulated in isolation; rather, they are organized through specific training methods, strategies, and systems that define how the stimulus is structured within and across training sessions [9]. Historically, methods such as drop sets, rest–pause, pyramidal loading, and pre-exhaustion have been widely employed with the intention of increasing muscular fatigue, metabolic stress, or perceived training intensity [7,10,11,12]. These approaches largely originated from empirical practice, particularly within bodybuilding and strength training communities, where experiential knowledge guided the evolution of training practice. However, despite their widespread adoption, the physiological rationale underpinning these methods has often been assumed rather than systematically validated, raising questions regarding their purported superiority over more traditional training structures [13].

Recent evidence synthesis has refined the understanding of resistance training prescription by identifying the variables that most consistently influence adaptation. Analyses indicate that hypertrophy is strongly associated with higher weekly training volumes, typically exceeding 10 sets per muscle group, whereas strength gains are optimized with higher relative loads (≥80% 1RM) and sufficient training frequency (≥2 sessions per week) [5,14,15]. Furthermore, progressive overload and appropriate periodization remain fundamental principles for promoting continued adaptation over time [15]. The relevance of proximity to muscular failure may depend on relative load, training volume, training status, and the outcome assessed [16,17]. Although momentary muscular failure is not necessary under all conditions, closer proximity to failure may be beneficial in specific contexts, particularly when low loads are used, while also increasing fatigue and recovery demands.

These findings have important implications for how resistance training methods, strategies, and systems are interpreted. Rather than acting as independent drivers of adaptation, methods are better understood as specific ways of delivering a training stimulus through the manipulation of foundational prescription variables. A training strategy operates at a higher level, governing why and when one or more methods are selected to achieve a defined objective under specific constraints. A training system, in turn, represents the broader framework through which methods and strategies are organized, sequenced, and progressed over time to support long-term adaptation. This perspective shifts the focus from identifying a universally superior method to understanding how different approaches may be selected and combined according to training goals, time availability, training status, recovery capacity, and long-term adherence.

Consistent with the narrative design, the historical, conceptual, and theoretical discussion was not restricted by publication date and integrated seminal studies, previous systematic reviews, and meta-analyses. The 2021–2026 window applied only to the focused descriptive mapping of recent primary experimental studies and was selected to provide a focused snapshot of approximately the most recent five years of experimental research available at the time of the search. This period was considered appropriate for characterizing contemporary study designs, populations, interventions, outcomes, and research trends, rather than for defining the historical development or complete evidence base of resistance training approaches. Concepts included within the historical and theoretical framework were therefore not required to be represented among the 40 studies included in the contemporary mapping and should be interpreted separately from the evidence summarized in Table 3. This temporal boundary should consequently not be interpreted as a physiological, historical, or conceptual threshold. Unlike previous evidence syntheses that primarily examined isolated prescription variables or comparisons between individual methods, the present review provides a conceptual and applied framework for distinguishing methods, strategies, and systems and for understanding how they operate at different levels of resistance training prescription. Accordingly, the purpose of this narrative review was to examine how these constructs organize and deliver established training variables under different goals and practical constraints, while integrating foundational, mechanistic, and contemporary evidence. A secondary objective was to characterize how these approaches have been investigated in primary experimental studies published between 2021 and 2026.

## 2. Materials and Methods

This narrative review comprised two complementary components. First, an unrestricted narrative synthesis was conducted to establish the historical background, conceptual framework, theoretical rationale, and broader interpretation of resistance training methods, strategies, and systems. No publication-date restriction was applied to this component, and seminal primary studies, systematic reviews, meta-analyses, umbrella reviews, and relevant narrative reviews were considered.

Second, an illustrative descriptive mapping of contemporary primary experimental evidence was conducted. PubMed, Scopus, and Web of Science were searched for studies published between January 2021 and April 2026. The final database search was conducted in April 2026. Google Scholar, backward reference screening, forward citation tracking, and Connected Papers were used as Supplementary Sources. The January 2021–April 2026 period was selected as an operational boundary to characterize the identified mapping domains (i.e., study designs, populations, interventions, outcomes, and research trends); it was not intended to define the complete evidence base of the review or to represent a physiological, historical, or conceptual threshold.

The search strategy was adapted to the syntax and search fields of each database using the following combination: (“resistance training” OR “strength training methods”) AND (“drop set” OR “rest–pause” OR “pyramidal training” OR “cluster sets” OR “German volume training” OR “circuit training” OR “blood flow restriction” OR “velocity-based training” OR “pre-exhaustion” OR “eccentric training”) AND (“hypertrophy” OR “strength” OR “muscular endurance” OR “power” OR “neuromuscular performance” OR “metabolic responses” OR “perceptual responses”), as presented in Supplementary File Table S1. Primary experimental studies were eligible for inclusion in Table 3 when they involved adults without diagnosed clinical conditions or musculoskeletal injuries that would substantially affect resistance training participation. Sedentary, untrained, recreationally active, resistance-trained, and middle-aged adults were eligible when they otherwise met this definition of health. Eligible studies were also required to clearly describe the resistance training method or set configuration and report at least one relevant physiological, perceptual, or performance outcome. Both longitudinal interventions and acute crossover studies were considered. Study selection and data extraction were performed independently by two authors. Disagreements regarding study eligibility or data extraction were resolved through discussion and consensus. Because the search was not conducted as a formal systematic screening process, detailed record counts at each stage were not prospectively documented.

No structured study-level risk-of-bias assessment was performed. Instead, the evidence was interpreted qualitatively, with attention paid to study design, sample size, intervention duration, participant characteristics, comparator conditions, outcome measures, consistency of findings, and methodological limitations. The contemporary mapping was intended to characterize recent research and outcome trends rather than to generate an exhaustive inventory of eligible studies or support evidence-graded comparative effectiveness conclusions.

Studies published before 2021 and secondary evidence sources were not included in the contemporary mapping presented in Table 3. However, they were used without temporal restriction throughout the narrative synthesis to support the historical context, conceptual framework, mechanistic interpretation, and discussion of the recent experimental findings. Given the narrative design, the broader evidence base was synthesized qualitatively, with attention to the direction and magnitude of findings, consistency across studies, methodological limitations, and mechanistic plausibility. The primary experimental studies included in the contemporary mapping were descriptively characterized according to study design, participant characteristics, training method, manipulated prescription dimension, assessed outcomes, and principal findings. No quantitative pooling or meta-analysis was performed.

## 3. Resistance Training Methods and Systems

Within the proposed framework, a resistance training method refers to the specific way in which the training stimulus is delivered or manipulated, whereas a strategy refers to the purposeful selection and application of one or more methods according to a defined goal or practical constraint. A training system represents a higher-order framework through which methods and strategies are organized and sequenced, and progress over time.

**Figure 1:**
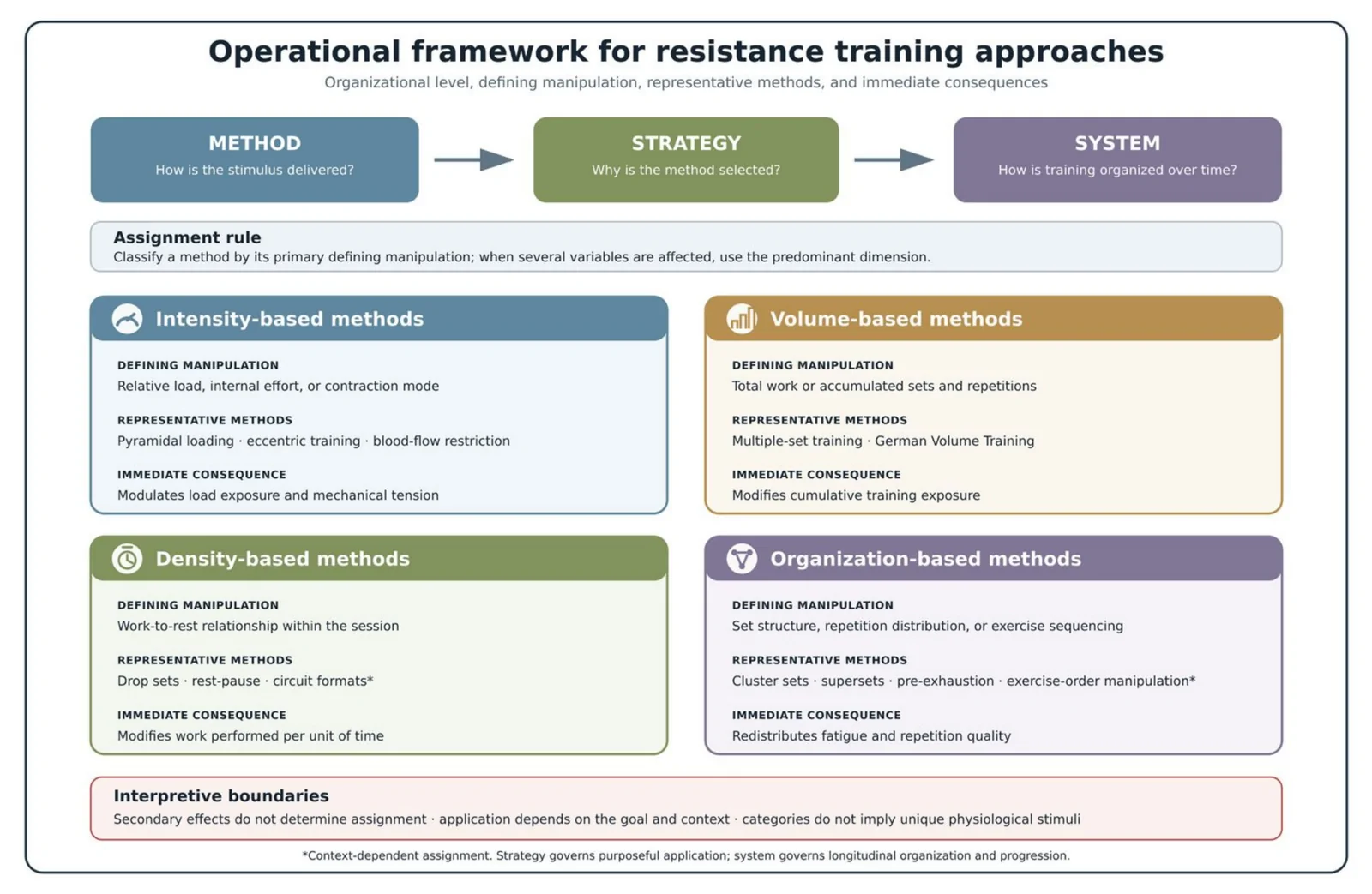
Operational classification of resistance training methods, strategies, and systems.

Training methods can be further classified according to the dimension of the stimulus they primarily manipulate: intensity, volume, density, or organization. These categories are not mutually exclusive and do not imply that each method produces a distinct physiological stimulus. Rather, the framework clarifies how different methods distribute established prescription variables and how their selection may influence training efficiency, fatigue distribution, movement velocity, and repetition quality. The operational relationships among resistance training methods, strategies, and systems are illustrated in Figure 1.

### 3.1. Methods Based on the Manipulation of Intensity

Methods that primarily manipulate intensity influence the magnitude and distribution of mechanical tension, which remains a primary determinant of strength development and an important contributor to hypertrophy. Mechanical tension directly affects motor unit recruitment, particularly the activation of high-threshold motor units associated with type II muscle fibers, and contributes to force-dependent intracellular signaling pathways [18]. Contemporary loading recommendations continue to support the use of higher loads for maximizing strength, whereas a broad loading spectrum appears capable of promoting hypertrophy when effort and weekly training volume are sufficient.

Pyramidal training is one of the most traditional examples of intensity manipulation. It typically involves progressive increases or decreases in load across successive sets, accompanied by inverse changes in repetition number. The theoretical rationale includes progressive motor unit recruitment, neural preparation for heavier efforts, and exposure to multiple loading zones within a single session [19]. Nevertheless, current evidence does not support a clear superiority of pyramidal loading over simpler fixed-load, multiple-set structures when the overall training stimulus is comparable. Its primary practical value appears to be organizational, allowing sessions to be structured around progressive exposure to heavier loads without implying a distinct adaptive mechanism.

Evidence comparing eccentric and concentric training remains mixed. Although no clear overall difference in muscle hypertrophy has been identified, subgroup findings suggest potential advantages of eccentric training for upper-limb muscles, shorter interventions, and outcomes assessed using specific measurement methods [20]. Eccentric-focused training nevertheless represents a distinct manipulation of intensity because eccentric force capacity exceeds concentric force capacity. Consequently, eccentric training can expose muscle tissue to greater force and mechanical strain, potentially enhancing strength adaptations, including concentric strength [21]. Recent reviews suggest that eccentric training may produce slightly greater strength gains than traditional training, whereas hypertrophy outcomes are generally similar when the overall training dose is comparable [22]. However, eccentric overload may increase muscle soreness, exercise-induced muscle damage, and recovery demands, which can limit its frequent implementation within periodized training programs [23].

Blood flow restriction (BFR) training represents a distinct form of intensity manipulation, as it increases internal effort and metabolic stress while utilizing low external loads, typically in the range of 20–40% 1RM. Recent meta-analyses indicate that low-load BFR can promote hypertrophy and muscle-related adaptations that are often comparable to those achieved with conventional higher-load resistance training [24], although maximal strength gains tend to be smaller than those achieved with high-load resistance training. Its effects may also vary according to participant characteristics and training status. Evidence in resistance-trained individuals remains limited and heterogeneous, whereas BFR may be particularly useful for untrained individuals, older adults, or populations that are unable to tolerate high mechanical loads. Accordingly, BFR is best interpreted as a complementary method that allows for meaningful adaptations under low-load conditions, rather than as a replacement for heavy-load training in strength-oriented programs. Conversely, new trends have been proposed during recent years, focusing on a low load complemented with neuromuscular electrostimulation [25].

Velocity-based training (VBT) uses movement velocity to prescribe and adjust training loads in real time. Its primary contribution is not necessarily to create a distinct physiological stimulus, but rather to improve load autoregulation, fatigue management, and repetition quality. Systematic reviews suggest that VBT can produce similar or, in some contexts, slightly superior improvements in strength and power compared with traditional percentage-based approaches, particularly when fatigue is managed using moderate velocity-loss thresholds [26]. Thus, VBT should be interpreted as a monitoring and prescription framework that refines the manipulation of intensity, rather than as a standalone method of adaptation. The primary rationale and evidence-based interpretation of these intensity-oriented methods are summarized in Table 1.

**Table 1:** Representative evidence for methods primarily manipulating intensity.

Method	Primary Rationale	Evidence-Based Interpretation
Pyramidal loading	Progressive exposure to multiple loading zones	Useful for session organization; no clear advantage over fixed-load training has been detected when the overall training stimulus is matched
Eccentric training	Greater force production and mechanical tension	May provide specific strength and architectural adaptations; no clear overall hypertrophy advantage has been established, and recovery demands may be greater
Blood flow restriction	Increased internal stress at low external loads	May be useful when high loads are not feasible; effects vary according to population and training status, and maximal strength outcomes generally favor high-load training
Velocity-based training	Real-time autoregulation of load and fatigue	Useful for performance monitoring and fatigue management; current evidence does not establish consistently superior long-term adaptations

### 3.2. Methods Based on the Manipulation of Volume and Training Density

Methods that manipulate both volume and density are particularly relevant in contemporary practice, as they are often promoted as time-efficient alternatives to traditional training structures [17,27]. Their primary advantage lies in increasing the number of effective repetitions or the total amount of work performed within a shorter session, rather than in generating a fundamentally distinct physiological stimulus. Accordingly, their utility is primarily practical, reflecting improvements in training efficiency rather than inherent differences in adaptive potential.

Drop set training extends a set through one or more immediate reductions in load following concentric failure or near failure. This structure increases local fatigue, prolongs effort, and typically reduces total session duration. Recent systematic reviews and meta-analyses indicate that drop sets can produce hypertrophy and strength outcomes that are broadly comparable to those achieved with traditional training [7], particularly when the overall work performed is similar. Accordingly, their primary practical value appears to lie in improving session efficiency rather than reflecting a distinct or superior adaptive mechanism.

Rest–pause training manipulates density by incorporating brief intra-set rest intervals, typically 10–30 s, allowing the continuation of repetitions with the same or a similar load [28]. This configuration may combine relatively high mechanical tension with reduced training time. Available evidence indicates that rest–pause training produces overall chronic outcomes like those of traditional training, although some studies suggest modest advantages in specific strength-related variables or localized muscular endurance under certain conditions [28]. As with drop sets, the primary benefit of rest–pause appears to be the efficient accumulation of effective repetitions rather than a distinct hypertrophic mechanism.

Supersets are training methods classified primarily within the organizational dimension because their defining feature is the pairing and sequencing of two exercises performed with minimal or no rest. Their effect on training density is a secondary consequence of this organization. Agonist–antagonist supersets may allow for reasonable preservation of performance while reducing rest intervals, whereas same-muscle supersets tend to increase local fatigue and muscular damage more substantially [29]. Accordingly, their primary value is practical, particularly for individuals with limited time availability, rather than reflecting a distinct or superior adaptive mechanism.

Circuit training represents a broader manipulation of training density and exercise organization, typically involving multiple exercises performed consecutively with minimal rest. Resistance-based circuit training has demonstrated utility for improving general fitness, body composition, and muscular endurance [30], and may also support strength gains in certain populations [31]. However, compared with more traditional resistance training structures, its efficacy for maximizing hypertrophy and maximal strength is limited by lower load exposure and greater interference from cumulative fatigue, particularly when cardiovascular demand is emphasized.

Within the context of contemporary recommendations, high-density methods are best understood as strategies that optimize the efficiency of training prescription rather than altering the fundamental biological determinants of adaptation. However, their greater fatigue cost necessitates careful programming to avoid compromising training frequency, recovery, and long-term progression. Consequently, their application should be strategically integrated within periodized programs to balance efficiency with sustainability of training adaptations. The main acute consequences and chronic interpretations of methods that primarily manipulate volume and density are summarized in Table 2.

**Table 2:** Representative evidence for methods primarily manipulating volume and density.

Method	Main Acute Consequence	Main Chronic Interpretation
Drop sets	High fatigue, short session, extended effort	No clear between-condition differences in overall strength or hypertrophy have been detected; drop sets may reduce session duration
Rest–pause	Brief recovery with repeated mini-bouts	No clear between-condition differences in overall chronic outcomes have been detected, although selected strength or muscular-endurance advantages may occur
Circuit training	High density and mixed metabolic demand	May improve general conditioning and muscular endurance; evidence is less consistent for maximizing hypertrophy or high-load strength

### 3.3. Contemporary Experimental Evidence: Study Characteristics and Reported Findings

**Figure 2:**
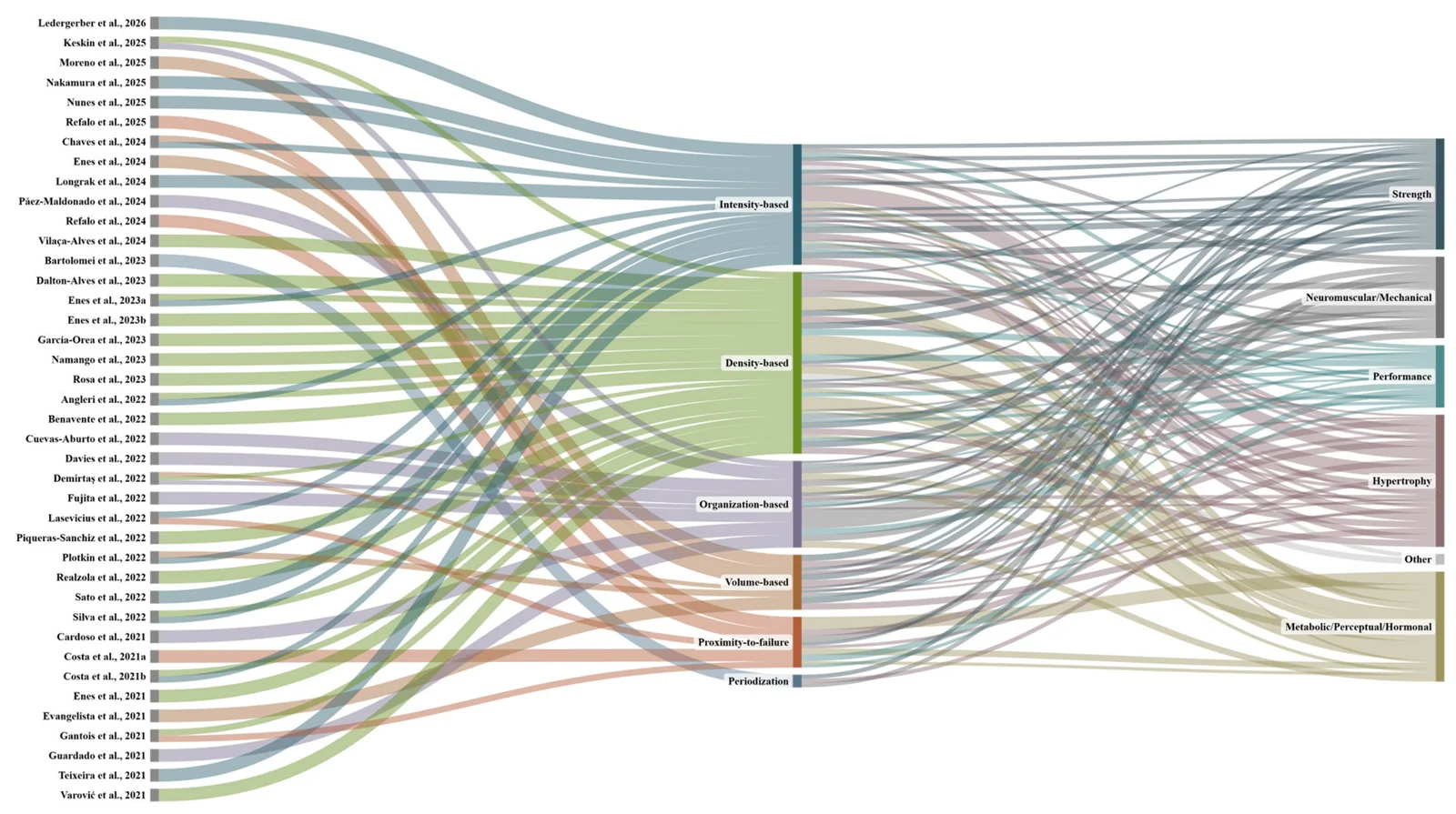
Sankey diagram of primary experimental studies included in the focused contemporary mapping [32,33,34,35,36,37,38,39,40,41,42,43,44,45,46,47,48,49,50,51,52,53,54,55,56,57,58,59,60,61,62,63,64,65,66,67,68,69,70,71], published between January 2021 and April 2026, linking the main resistance training methods and strategies to the primary outcomes assessed. Note: Flow widths are proportional to the number of studies; when a study included multiple methods or outcomes, its contribution was divided proportionally to maintain a total weight of one study.

To characterize contemporary experimental research, 40 primary studies published between January 2021 and April 2026 were descriptively mapped. This focused sample was used to examine recent resistance training methods, outcomes, and key findings and should not be interpreted as the complete historical evidence base considered in this narrative review. Regarding training methods, density-based approaches were most frequent (n = 18 [32,33,34,35,36,37,38,39,40,41,42,43,44,45,46,47,48,49]), followed by intensity-based (n = 13 [32,34,38,47,50,51,52,53,54,55,56,57,58]), organization-based (n = 8 [36,42,59,60,61,62,63,64]), volume-based (n = 6 [36,50,56,65,66,67]), and proximity-to-failure methods (n = 5 [40,51,68,69,70]). Periodization was investigated in one study [71]. Adaptations were assessed primarily through hypertrophy (n = 21 [32,36,37,39,42,48,49,50,51,53,54,55,56,57,58,61,65,66,67,69,71]) and strength (n = 20 [32,36,37,39,41,42,48,50,51,52,54,55,56,57,58,61,65,66,67,71]). Neuromuscular/mechanical outcomes (n = 14 [34,35,44,47,52,55,59,60,61,62,63,64,69,71]) and metabolic/perceptual/hormonal outcomes (n = 14 [33,35,38,40,43,44,45,46,47,60,63,64,68,70]) were each evaluated in 14 studies. Performance outcomes were assessed in 12 studies (n = 12 [34,40,41,42,44,46,47,52,56,59,61,68]), whereas other outcomes were assessed in two studies (n = 2 [33,54]). The relationships between the resistance training methods and strategies investigated and the primary outcomes assessed are summarized in Figure 2.

Within the focused 2021–2026 experimental sample, the studies summarized in Table 3 provide a descriptive overview of the muscular adaptations reported across different resistance training methods and strategies. These study-level observations were interpreted alongside the unrestricted historical, conceptual, and synthesized literature discussed throughout the review and should not be considered the complete evidence base for resistance training methods or as evidence of comparative superiority between methods.

**Table 3:** Characteristics and findings reported within each of the 40 experimental studies included in the contemporary mapping (2021–2026).

Author (Year)	Aim	Method or Strategy of Training	Outcomes	Findings Reported Within Each Study
Ledergerber et al., 2026 [52]	To compare changes in voluntary activation (VA) and neuromuscular performance following 8 weeks of low-load blood-flow restriction (BFR) versus high-load (HL) knee extensor training in middle-aged adults, and to examine the effects of a subsequent 2-week HL phase in both groups.	BFR HL	Neuromuscular Strength Mechanical/Performance	Voluntary activation/Strength/RFD: HL > BFR Jump performance: BFR = HL
Refalo et al., 2025 [70]	To investigate the effect of proximity-to-failure on perceptual responses (perceived discomfort, exertion, and general feelings) during and following resistance training.	FAIL RIR	Perceptual (discomfort/exertion/valence)	Perceptual load: FAIL > RIR
Moreno et al., 2025 [67]	To compare the effects of prescribing an increased number of sets relative to baseline (ITV) to a maintenance of baseline training volume (BTV) in previously trained individuals.	Increased sets TR Maintenance sets TR	Hypertrophy Strength	Hypertrophy and Strength: Increased sets TR = Maintenance sets TR
Nakamura et al., 2025 [54]	To test the hypothesis that inclusion of prolonged eccentric contractions would increase muscle adaptations greater than concentric-only contractions.	CON-ECC CON ECC	Strength Hypertrophy (MT) Range of motion	Torque/MT/Range of motion: CON-ECC > CON
Keskin et al., 2025 [42]	To investigate the impact of equated volume load on three different RT methods (traditional, pre-exhaustion, and drop sets) on muscle strength, endurance, and hypertrophy in young men.	DS TR Pre-exhaustion	Strength Hypertrophy Muscular endurance	Strength, hypertrophy and muscular endurance using volume equated: TR = Pre-exhaustion = DS
Nunes et al., 2025 [55]	To compare the effects of concentric (CON-RT) and eccentric (ECC-RT) resistance training matched for relative intensity and work on regional hypertrophy, muscle architecture, and function, and to explore associations between outcomes.	ECC CON	Hypertrophy (MT) Muscle architecture (pennation angle, fascicle length) Strength	MT: CON ≈ ECC Pennation angle: CON > ECC Fascicle length: ECC > CON Torque gains: ECC > CON
Longrak et al., 2024 [53]	To compare regional morphological adaptations of the vastus lateralis muscle in response to high-intensity traditional (HI) vs. mixed-intensity (MIX) progressive resistance training programs.	High-intensity TR Mixed-intensity TR	Regional hypertrophy	Hypertrophy: High-intensity TR = Mixed-intensity TR
Enes et al., 2024 [65]	To investigate the effect of progressively adding sets for the lower limb every 2 weeks versus performing a constant set volume in resistance-trained males.	Constant volume 4-set progression 6-set progression	Strength (1RM) Hypertrophy (MT)	Strength: 6-set progression > 4-set progression = Constant volume Hypertrophy: 6-set progression = 4-set progression = Constant volume
Chaves et al., 2024 [50]	To compare the effects of progressive overload via increasing load (LOADprog) versus increasing repetitions (REPSprog) on muscle strength and cross-sectional area (CSA).	LOADprog REPSprog	Strength Hypertrophy (CSA)	Strength and hypertrophy: LOAD = REPS
Vilaça-Alves et al., 2024 [49]	To investigate the effects of two strength training protocols (drop-set vs. traditional), equated in volume, on elbow flexor MT in women.	DS TR	Hypertrophy (elbow flexors)	MT: DS = TR > Control
Refalo et al., 2024 [69]	To examine the influence of RT proximity-to-failure, determined by repetitions-in-reserve (RIR), on quadriceps hypertrophy and neuromuscular fatigue following 8 weeks of RT performed to either momentary muscular failure or with RIR in resistance-trained individuals.	FAIL RIR	Hypertrophy (MT quadriceps) Mechanical fatigue	MT quadriceps: FAIL ≈ RIR Fatigue/velocity loss: FAIL > RIR
Páez-Maldonado et al., 2024 [64]	To examine the acute effects on mechanical, neuromuscular, metabolic, and muscle contractile responses to different set configurations (TR and CS) in full-squat	TR CS6 (30 s intra-set rest after 6th repetition) CS2 (30 s intra-set rest every 2 repetitions)	Mechanical performance Neuromuscular (EMG) Metabolic stress	Mechanical performance & fatigue resistance: CS2 > CS6 > TR Metabolic stress: CS2 = CS6 < TR
Enes et al., 2023a [38]	To compare the effects of drop-set, descending pyramid, and traditional resistance training on psychophysiological responses in men.	DS DP TR	Perceptual/Psychophysiological responses (RPE, Feelings of Pleasure/Displeasure)	Perceptual/psychophysiological using volume equated: DS > DP > TR
Namango et al., 2023 [43]	To assess the levels of plasma growth hormone (GH) and IGF during drop set and concentric exercises.	DS TR	Hormonal/Metabolic	Hormonal/metabolic: increase in both conditions
García-Orea et al., 2023 [41]	To compare the effect of two velocity-based training programs only differing in the set configuration on muscle strength, muscle endurance, and jump performance.	TR Alternating sets (paired exercises)	Performance Strength (1RM) Muscular endurance Training time/efficiency	Strength & performance: Alternating sets = TR Muscular endurance: Alternating sets > TR Training efficiency: Alternating sets more time-efficient
Enes et al., 2023b [39]	To investigate the effect of drop-set (DS) and rest–pause (RP) systems compared to traditional (TRAD) resistance training on muscular adaptations and psychophysiological responses.	DS RP TR	Strength (1RM) Hypertrophy (MT)	Strength: RP = TR > DS Hypertrophy: temporal increases, RP > TR = DS
Bartolomei et al., 2023 [71]	To compare the effects of 2 different periodized resistance training programs (mixed session vs. block periodization) on maximal strength, power, and muscle architecture in trained individuals.	Mixed session Block periodization	Strength (1RM) Hypertrophy (MT) Power	Strength and hypertrophy: Mixed session > Block periodization Power: Block periodization > Mixed session
Rosa et al., 2023 [46]	To compare the effects of four different rest–pause methods on total repetitions completed, RPE, and blood lactate responses during the leg press exercise.	RP—three rest interval lengths (1, 2, 3 min)	Performance (total reps) Perceptual Metabolic	Repetition performance: 1 min < 2 min = 3 min Blood lactate: Slightly higher with longer rest; BS > LE Discomfort (RPE): Longer rest = lower discomfort; LE < BS
Dalton-Alves et al., 2023 [35]	To test whether there are differences in neuromuscular, metabolic, and perceptual alterations in response to traditional and more frequent and shorter total rest configurations with similar intensity and volume	TR Frequent Shorter Rest (30 sec rest intervals between sets)	Neuromuscular Metabolic stress Perceptual (load)	Neuromuscular: Frequent Shorter Rest > TR Metabolic stress: TR > Frequent Shorter Rest Perceived load: TR > Frequent Shorter Rest
Sato et al., 2022 [57]	To compare CON-ECC, CON, and ECC resistance training of the elbow flexors for effects on muscle strength and hypertrophy.	CON-ECC CON ECC	Strength (isometric) Hypertrophy (MT)	Strength: CON-ECC and ECC increased Hypertrophy: CON-ECC ≈ ECC > CON
Fujita et al., 2022 [62]	To investigate the effects of the pre-exhaustion method on electromyographic activity at different interval sets.	TR Pre-exhaustion TR	Neuromuscular (EMG)	Neuromuscular: Pre-exhaustion = No pre-exhaustion
Davies et al., 2022 [61]	To explore the effect of volume-equated traditional-set and cluster-set structures on upper-body muscular hypertrophy and performance after high-load resistance training.	TR CS	Hypertrophy Strength Performance Mechanical	Mechanical: TR > CS Hypertrophy: TR > CS Strength and performance: TR = CS
Plotkin et al., 2022 [56]	To compare the effects of increasing load while keeping repetitions constant (LOAD) vs. increasing repetitions while keeping load constant (REPS) on lower body hypertrophy, strength, and endurance.	LOADprog REPSprog	Strength Hypertrophy Endurance	Hypertrophy: REPSprog > LOADprog Strength and endurance: LOADprog > REPSprog
Silva et al., 2022 [47]	To evaluate and compare the metabolic and physiological responses of traditional, drop set, and blood flow restriction (BFR) training methods.	BFR DS TR	Performance Mechanical Perceptual	Mechanical: BFR ≈ DS Performance: DS > others Perceived exertion: DS > others
Demirtaş et al., 2022 [36]	To compare the effects of Modified German Volume Training (MGVT), Super Set (SS), and Giant Set (GS) on strength and hypertrophy values.	MGVT SS GS	Strength (1RM) Hypertrophy (CSA)	Strength and hypertrophy: MGVT = SS = GS (all groups increased)
Benavente et al., 2022 [33]	To examine the effects of moderate altitude exposure combined with resistance training on muscle hypertrophy and strength gains compared to sea-level training in trained men.	TR (altitude) + Rest intervals (60 s vs. 120 s) TR (sea level) + Rest intervals (60 s vs. 120 s)	Metabolic Cardiovascular (recovery) Perceptual	Hypertrophy: TR (altitude) ≈ TR (sea level) Metabolic stress: 60 s rest > 120 s rest (all conditions) Recovery: TR (altitude) > TR (sea level)
Angleri et al., 2022 [32]	To determine whether resistance training variable manipulations (different set configurations) are less important than individual intrinsic biological factors in promoting muscle fiber hypertrophy.	DS CP TR	Hypertrophy (fiber CSA) Strength (1RM)	Individual biology explains variance > protocol. Hypertrophy (CSA): DS = CP = TR Strength (1-RM): DS = CP = TR
Lasevicius et al., 2022 [51]	To compare the effects of training to muscle failure versus non-failure on muscle hypertrophy and strength adaptations using both high-load and low-load resistance training protocols.	HL (FAIL vs. no FAIL) LL (FAIL vs. no FAIL)	Hypertrophy Strength	LL: Failure > Non-failure (hypertrophy) HL: Failure ≈ Non-failure Strength: increased in all 1RM: HL > LL
Realzola et al., 2022 [45]	To compare the metabolic profile (oxygen consumption, blood lactate, energy expenditure, excess post-exercise oxygen consumption) of reciprocal supersets versus traditional resistance training in recreationally active adults.	SS TR	Metabolic (oxygen consumption, lactate, excess post-exercise oxygen consumption)	Metabolic: SS > TR
Piqueras-Sanchiz et al., 2022 [44]	To investigate the effect of set configuration on mechanical performance, neuromuscular activity, metabolic response, and muscle contractile properties	TR 3 × 8 (long rest) TR 6 × 4 (shorter rest)	Mechanical Neuromuscular (EMG) Metabolic (lactate, ammonia) Performance	Mechanical performance & fatigue resistance: TR 6 × 4 > TR 3 × 8 Metabolic stress: 3 × 8 > 6 × 4
Cuevas-Aburto et al., 2022 [60]	To compare the effects of traditional, cluster, and rest redistribution set configurations on kinetic and kinematic outputs, mechanical fatigue, and perceptual responses during the bench press exercise.	TR CS Rest-redistribution	Mechanical (bar velocity) Perceptual (RPE)	Mechanical: CS > Rest-redistribution > TR Perceptual: TR = CS = Rest-redistribution (both reduce)
Cardoso et al., 2021 [59]	To compare different rest intervals between agonist–antagonist actions during agonist–antagonist paired-sets (APS) in young adults.	APS—0 s between actions APS—60 s between actions APS—120 s between actions	Performance Neuromuscular	Performance & neuromuscular: APS—0 s = APS—60 s = APS—120 s Neuromuscular fatigue: APS—60 s > APS—0 s = APS—120 s
Costa et al., 2021a [68]	To analyze the acute effects of repetitions to failure (RF) or not to failure (RNF) on CMJ performance and internal training load in trained male adults.	FAIL no FAIL	Performance Perceptual	Performance: FAIL = no FAIL Perceived exertion: FAIL > no FAIL
Costa et al., 2021b [34]	To analyze the acute effects of RT systems (drop-set, decrescent pyramid, and traditional) on lower- and upper-limb performance in trained adults.	DS DP TR	Performance (lower and upper limbs) Neuromuscular fatigue (lower and upper limbs)	Performance and neuromuscular fatigue: Lower-limb fatigue: DS = DP > TR Upper-limb fatigue: DS > DP = TR
Gantois et al., 2021 [40]	To compare the acute effects of traditional and rest–pause resistance exercise done to muscular failure on CMJ performance.	TR-FAIL TR-no FAIL RP-FAIL RP-no FAIL	Performance impairment Metabolic (blood lactate) Perceptual	Performance impairment: RP-FAIL > all others (highest acute fatigue) Metabolic: TR-FAIL & RP-FAIL > TR-no FAIL & RP-no Fail Perceived load: FAIL > No FAIL
Evangelista et al., 2021 [66]	To investigate the effects of different RT repetition schemes with equalized volumes on muscle adaptations.	Volume equated (set × rep): 10 × 3 3 × 10 5 × 6	Strength (1RM) Hypertrophy (MT)	Strength & hypertrophy: 10 × 3 = 3 × 10 = 5 × 6 (all increased)
Varović et al., 2021 [48]	To compare the effects of drop set RT versus traditional RT on maximal muscle strength and regional hypertrophy of the quadriceps femoris.	DS TR	Hypertrophy (MT) Strength (1RM)	Hypertrophy: DS > TR (both increased) Strength: DS = TR
Guardado et al., 2021 [63]	To compare the perceptual responses, physiological indicators, and technical parameters between different cluster training configurations for upper body exercises.	TR CS1 (4 × 2 + 2+2 reps; 15 s + 80 s) CS2 (24 reps + 15 s)	Metabolic (bar velocity) Perceptual Mechanical	Mechanical: CS1 = CS2 > TR Perceptual & metabolic: TR > CS1 = CS2
Enes et al., 2021 [37]	To compare DS and RP systems versus traditional training with equalized total training volume on 1RM and thigh MT.	DS RP TR	Strength (1RM) Hypertrophy (MT)	Strength: RP > DS = TR Hypertrophy: RP = DS = TR (all groups increased)
Teixeira et al., 2021 [58]	To determine whether adding blood flow restriction (BFR) exercise as a recovery strategy after high-intensity resistance training promotes additional muscle hypertrophy and strength gains.	BFR during rest intervals + HL-RT BFR during muscle contractions + HL-RT HL-RT alone (no BFR)	Hypertrophy	Strength & hypertrophy: BFR during rest intervals = BFR during muscle contractions = HL alone Metabolic stress ([La]): BFR during rest intervals > BFR during muscle contractions = HL alone
			Strength	

Notes: 1RM—One repetition maximum; VA—voluntary activation; RFD—rate of force development; ITV—increased training volume; BTV—baseline training volume; MT—muscle thickness; RT—resistance training; CS—cluster set; TRAD—traditional resistance training; BS—back squat; LE—leg extension; RPE—rating of perceived exertion; CMJ—countermovement jump; APS—Agonist–antagonist paired sets; BFR—Blood flow restriction; CONC—Concentric-only resistance training; CP—crescent pyramid; CSA-cross-sectional area; DS—Drop-set; DP—decrescent pyramid; ECC—Eccentric-only resistance training; EMG—electromyography; FAIL—Failure; GS—Giant Set; HL—High-load resistance training; LL—Low-load resistance training; LOADprog—Load progression;; MGVT—Modified German Volume Training; MT—muscle thickness; REPSprog—Repetition progression; RIR—Repetitions-in-reserve; RP—Rest–pause set; SS—Super Set; TR—Traditional set. The symbols “>” and “<” reproduce the direction of the differences reported within each individual study and should not be interpreted as evidence of the general superiority or inferiority of one resistance training method over another. The symbols “=” and “≈” indicate that no statistically significant between-condition difference was reported and should not be interpreted as evidence of statistical equivalence. Effect sizes and confidence intervals were not consistently reported across the original studies, limiting interpretation of the magnitude and certainty of the findings.

Across several controlled comparisons included in the descriptive mapping, individual studies reported no statistically significant between-condition differences for selected strength or hypertrophy outcomes [42,49,50,67]. However, these findings should not be interpreted as evidence of true equivalence, because the studies involved relatively small samples and did not employ equivalence or non-inferiority designs. Other individual studies reported context-specific differences in training efficiency [41], fatigue distribution and preservation of movement velocity [44,60,63,64], regional hypertrophy [48], and contraction-specific strength or architectural adaptations [54,55,57]. In two studies, higher-load conditions were associated with greater improvements in selected maximal-strength outcomes than lower-load conditions [51,52], although these observations should not be generalized beyond the specific populations, protocols, and outcomes examined.

Individual studies also reported differences in selected secondary and outcome-specific variables. Within specific comparisons, eccentric or combined concentric–eccentric training was associated with differences in muscle architecture and contraction-specific strength, including greater fascicle-length or torque gains, although these effects were not consistently accompanied by greater overall hypertrophy [54,55,57]. Similarly, several acute studies reported greater preservation of movement velocity or lower mechanical or perceptual fatigue with cluster-set and rest-redistribution configurations than with traditional continuous sets [44,60,63,64]. Studies examining proximity to failure reported that momentary muscular failure may not be necessary for hypertrophy under all conditions, whereas training to failure may increase fatigue, discomfort, and velocity loss [40,46,51,68,69,70]. Taken descriptively, these findings suggest that methods associated with similar primary adaptations may nevertheless differ in selected context-specific outcomes, including fatigue cost, repetition quality, regional or architectural responses, and practical applicability. These observations should not be interpreted as establishing the general superiority of any method.

The methodological characteristics of the included evidence should be considered when interpreting these findings. Fourteen studies examined acute responses to a single resistance training session, whereas 26 used longitudinal or intervention designs, generally lasting 5–12 weeks. Sample sizes were relatively small, ranging from 11 to 53 participants, and the studies differed in participant training status, comparator conditions, outcome measures, and the extent to which training volume and intensity were matched. Acute crossover studies provide information about immediate mechanical, neuromuscular, metabolic, and perceptual responses but cannot establish chronic adaptations. Moreover, the short duration and small samples of several longitudinal studies restrict conclusions regarding long-term effectiveness and inter-individual variability. Because no formal risk-of-bias or certainty-of-evidence assessment was performed, the methodological quality and evidential weight of individual studies cannot be formally compared. The findings of the contemporary mapping should therefore be interpreted as descriptive and exploratory rather than as definitive or evidence-graded conclusions.

## 4. Future Directions

Future research should prioritize long-term resistance training interventions, particularly those extending beyond six months and involving trained individuals, to determine whether the absence of clear between-method differences in selected outcomes persists under conditions of sustained overload and accumulated fatigue. More standardized operational definitions are also needed to ensure that methods such as drop sets, rest–pause, supersets, circuit training, BFR, and cluster sets are genuinely comparable across studies. Furthermore, within the contemporary experimental sample mapped between 2021 and 2026, studies were predominantly focused on traditional, drop-set, rest–pause, and BFR approaches, whereas cluster sets, pre-exhaustion, VBT, and eccentric-focused strategies received comparatively less longitudinal investigation.

Another important direction involves the investigation of inter-individual variability in response to different methods. Training status, fiber-type profile, recovery capacity, psychological tolerance to effort, and time availability may all influence how a given individual responds to a particular training structure. Moving beyond group averages may help identify whether certain methods are more effective under specific contexts or populations. A meaningful conceptual advance would be the development of an integrative framework that clearly distinguishes primary training variables from secondary organizational strategies. Within such a model, volume, intensity, frequency, effort, and progression would be positioned as primary drivers of adaptation, whereas methods would be interpreted as modulators that shape how these variables are distributed within and across training sessions.

Future studies should examine whether time-efficient methods such as drop sets and rest–pause produce similar adaptations when an equivalent weekly training volume is distributed across fewer, longer sessions or multiple shorter, microdosed sessions. Such comparisons should assess not only strength and hypertrophy but also adherence, fatigue, recovery, and session efficiency. Further longitudinal research is needed to determine whether high-load, eccentric, cluster-set, and velocity-based configurations provide specific benefits for rate of force development, movement velocity, and power-related outcomes relevant to explosive sports. None of the studies included in the contemporary mapping directly examined HYROX-style competition or comparable hybrid and concurrent training demands. Future research should therefore investigate whether time-efficient and fatigue-managed resistance training methods can preserve strength and power adaptations while reducing session duration, managing recovery demands, and minimizing potential interference with endurance training.

## 5. Practical Application

Resistance training methods should be deployed strategically, as a tool to manipulate volume, intensity, density, or repetition quality, rather than as independent stimuli. Their application should be aligned with the specific training goal and the practical constraints of the individual. For maximal strength development, traditional multiple-set training with high relative loads remains the most direct and robust approach. Cluster sets may be useful when the goal is to preserve repetition quality or bar velocity and force output across repetitions when managing intra-session fatigue. The use of VBT may further refine load prescription and fatigue management.

For hypertrophy, several methods can be effective provided that total weekly volume, effort, and progression are sufficient. Traditional multiple-set training remains a reliable option, whereas drop sets and rest–pause methods can increase training density and reduce session duration. German Volume Training (GVT) may be considered a high-volume variation, but it should not be assumed superior simply because it prescribes more sets. BFR training may be useful when low-load conditions are necessary, but it should generally be interpreted as a complementary rather than substitutive approach in strength-oriented contexts.

For power development and repetition quality, methods that preserve movement velocity should be prioritized. Cluster sets and lower-fatigue VBT prescriptions are therefore more appropriate than high-density methods such as extended drop sets or aggressive rest–pause sequences. By contrast, organizational methods such as supersets can also serve as time-efficient strategies for metabolic conditioning when preservation of peak power is not the primary objective.

Contemporary evidence indicates that resistance training prescription should prioritize adequate training volume, appropriate intensity, sufficient weekly frequency, and progressive overload, regardless of the specific method employed. Methods should be selected according to individual goals, training status, recovery capacity, and practical constraints, rather than being viewed as inherently superior approaches. Strategic variation of methods may also enhance adherence, reduce training monotony, and contribute to the long-term sustainability of resistance training programs. Accordingly, practical implementation should involve not only the selection of appropriate training methods, but also their strategic application within a coherent periodized system that governs sequencing, progression, recovery, and adjustment across the training cycle.

## 6. Conclusions

Through the integration of unrestricted foundational and synthesized literature with a focused mapping of contemporary primary experimental studies, it appears that resistance training adaptations are governed predominantly by foundational prescription variables rather than by the nominal method per se. Volume, intensity, frequency, effort, and progression remain the principal determinants of strength and hypertrophy adaptations. The lack of consistent robust between-method differences should not, however, be construed as evidence of true methodological equivalence. Individual studies reported method-specific differences for selected outcomes and contexts, including selected maximal-strength indicators in comparisons involving rest–pause training, regional hypertrophy in a specific comparison involving drop-set training, and outcomes such as torque, fascicle length, muscle architecture, and selected hypertrophy measures in comparisons involving eccentric or combined concentric–eccentric training. These observations should be interpreted as outcome- and context-specific, rather than as evidence of any universally superior method. Accordingly, resistance training methods are most appropriately conceptualized as organizational frameworks for established training variables that modulate session efficiency, fatigue distribution, internal load, repetition quality, and specific mechanical demands. Method selection should therefore be determined by the intended outcomes, individual athlete or patient characteristics, recovery capacity, and practical constraints, and should be embedded within a coherent, periodized training framework.

## Data Availability

No new data were created or analyzed in this study.

## Supplementary Materials

The following supporting information can be downloaded at: https://www.mdpi.com/article/10.3390/sports14080348/s1, Table S1. Complete Search Strategies for PubMed, Scopus, and Web of Science.

## Abbreviations

The following abbreviations are used in this manuscript:

1RM	One repetition maximum
AEL	Accentuated eccentric loading
APS	Agonist–antagonist paired sets
BFR	Blood flow restriction
BP	Block periodization
CON	Concentric-only resistance training
CSA	Cross-sectional area
DS	Drop-set
ECC	Eccentric-only resistance training
EMG	Electromyography
FAIL	Failure
GS	Giant Set
HL	High-load resistance training
LL	Low-load resistance training
LOADprog	Load progression
MGVT	Modified German Volume Training
MSP	Mixed session periodization
MT	Muscle thickness
REPSprog	Repetition progression
RIR	Repetitions-in-reserve
RP	Rest–pause set
SS	Superset
TR	Traditional set
VBT	Velocity-based training
