# Supplementary material for: Methods and Systems of Resistance Training: A Narrative Review Based on Contemporary Recommendations
Source: Sports (Basel). 2026 Aug 11;14(8):348. doi: 10.3390/sports14080348 (PMC13517766; doi:10.3390/sports14080348)
Supplement: Supplementary file 1 [file sports-14-00348-s001.zip › sports-4425106-supplementary.pdf]

Supplementary Table S1. Complete Search Strategies for PubMed, Scopus, and Web of Science

Database	Search strategy
**PubMed**	("resistance training"[tiab] OR "strength training methods"[tiab]) AND ("drop set"[tiab] OR "rest-pause"[tiab] OR "pyramidal training"[tiab] OR "cluster sets"[tiab] OR "German volume training"[tiab] OR "circuit training"[tiab] OR "blood flow restriction"[tiab] OR "velocity-based training"[tiab] OR "pre-exhaustion"[tiab] OR "eccentric training"[tiab]) AND ("hypertrophy"[tiab] OR "strength"[tiab] OR "muscular endurance"[tiab] OR "power"[tiab] OR "neuromuscular performance"[tiab] OR "metabolic responses"[tiab] OR "perceptual responses"[tiab]) AND ("2021/01/01"[pdat] : "2026/04/30"[pdat])
**Scopus**	(TITLE-ABS-KEY("resistance training" OR "strength training methods") AND TITLE-ABS-KEY("drop set" OR "rest-pause" OR "pyramidal training" OR "cluster sets" OR "German volume training" OR "circuit training" OR "blood flow restriction" OR "velocity-based training" OR "pre-exhaustion" OR "eccentric training") AND TITLE-ABS-KEY("hypertrophy" OR "strength" OR "muscular endurance" OR "power" OR "neuromuscular performance" OR "metabolic responses" OR "perceptual responses")) AND PUBYEAR > 2020 AND PUBYEAR < 2027
**Web of Science**	TS=("resistance training" OR "strength training methods") AND TS=("drop set" OR "rest-pause" OR "pyramidal training" OR "cluster sets" OR "German volume training" OR "circuit training" OR "blood flow restriction" OR "velocity-based training" OR "pre-exhaustion" OR "eccentric training") AND TS=("hypertrophy" OR "strength" OR "muscular endurance" OR "power" OR "neuromuscular performance" OR "metabolic responses" OR "perceptual responses")
